# Sequence diversity and evolution of infectious bursal disease virus in Iraq

**DOI:** 10.12688/f1000research.28421.1

**Published:** 2021-04-16

**Authors:** Ali Hadi Abbas, Haider Abas AL saegh, Furkan Sabbar ALaraji

**Affiliations:** 1 Microbiology, Faculty of veterinary medicine, University of Kufa, Najaf, Iraq; 2 Pathology and poultry diseases, Faculty of veterinary medicine, University of Kufa, Najaf, Iraq

**Keywords:** infectious bursal disease, VP1 gene, VP2 gene, hvVP2, poultry viral diseases, Gumboro, RNA viruses, very virulent vvIBDV.

## Abstract

**Background: **Infectious Bursal Disease (IBD) is a highly infectious disease which causes huge economic losses to the poultry industry due to the direct impact of the illness and indirect consequences such as decreasing the general immunity of the flock, leaving it naive to other diseases. In Iraq, IBD is highly prevalent despite vaccination programs, yet studies on sequence diversity of the causative virus are still rare.

**Methods:** A sample from Bursa of Fabricius from an IBD outbreak in a flock in the city of Najaf in Iraq was smeared on an FTA card. Amplicons of targeted regions in VP1 and VP2 genes were generated and sequenced. Sequences were then compared with other local and global sequences downloaded from GenBank repositories. Sequence alignment and DNA sequence analyses were achieved using MUSCLE, UGENE and MEGAx software. The molecular clock and sequence evolutionary analyses were applied using MEGAx tools.

**Results:** The strain sequenced in this study belongs to a very virulent Infectious Bursal Disease Virus (vvIBDV) as the DNA and phylogenetic analysis of VP1 and VP2 gene sequences showed a mutual clustering with similar sequences belonging to vvIBDV genogroup 3. Analyses of the hyper variable region of VP2 gene (hvVP2) of IBDV isolates from Iraq indicates a presence of sequence diversity. Interestingly, the two vaccine strains Ventri IBDV Plus and ABIC MB71 that showed the highest sequence similarity to the local isolates in the hvVP2 region are not used in vaccination routine against IBDV in Iraq.

**Conclusion:** Sequences of vvIBDV in Iraq are diverse. Remarkably, some of the available vaccine strains show high sequence similarity with local strains in Iraq; however, they are not included in the routine vaccination programs. Analysis of more samples involving more geographical regions is needed to draw a detailed map of antigenic diversity of IBDV in Iraq.

## Introduction

Infectious Bursal Disease (IBD), or Gumboro, is a highly contagious disease of poultry, characterized by severe immune suppression ( Van Den Berg, 2000). The birds surviving IBD suffer from poor feed conversion rate, poor growth, decreased egg production and quality, and reduced efficiency of vaccines. The economic losses due to IBD are not limited to the direct effect of the diseases, but also from decreasing the overall immune status of the flock leading it to be vulnerable to other diseases characterized by a high mortality rate such as Newcastle disease and Infectious Bronchitis. This immune suppression is a result of damage of Bursa of Fabricius, the target of IBDV, causing severe suppression to humoral and cellular immunity in the early stages of the bird’s life ( Rautenschlein *et al.*, 2002).

The causative agent of Infectious Bursal Disease (IBD) belongs to a viral family called *Birnaviridae* genus *avibirnavirus*, characterized by a double-stranded RNA viral genome consisting of two segments: the larger segment A and the smaller one B ( Dobos *et al.*, 1979). Segment A, known to contain the open reading frame (ORF), encodes for the capsid protein that comprises the epitope, which in turn interact with protective antibodies ( By Kevin J. Fahey, 1989; von Einem *et al.*, 2004; Coulibaly *et al.*, 2005), while segment B hosts the ORF VP1, which is responsible for viral replication and, hence, pathogenicity of the virus ( von Einem *et al.*, 2004).

This virus is prone to frequent genomic recombination events, genetic reassortments of the RNA segments, and mutations that could allow changes in the virulence and most likely the antigenicity of the virus ( Jackwood *et al.*, 2008; Ibdv, Jackwood and Sommer-wagner, 2011).

Infectious Bursal Disease Virus (IBDV) serotype1 is the most important serotype and it can be classified into three subgroups according to their virulence: sub-clinical (scIBDV), classical virulent (cvIBDV) and vey virulent (vvIBDV) ( Berg *et al.*, 2004). However, antigenic drift, shift and even a single nucleotide polymorphism (SNP) have shown to play a major role in creating antigenically different subtypes. These changes have been found to affect a specific region in the *VP2* gene called hyper variable region (hvVP2) ( Bayliss *et al.*, 1990; Eterradossi *et al.*, 1997, 1998; Brandt *et al.*, 2001).

Despite the vaccination programs that have been applied across the world, outbreaks in poultry flocks are still frequent, making IBD one of the most important diseases that hampers the poultry industry worldwide. In Iraq, although vaccination is practiced, the infection rate is high. Furthermore, studies on sequence diversity of the genes *VP1* and *VP2,* as well as the link between the hvVP2 region of local strains with available vaccine strains, are limited. Here, we analysed the pathogenicity and the sequence diversity of the antigenic determinant (VP1 and hvVP2) of an isolated strain from Najaf city and all other sequences from Iraq isolated from other regions of the country available in GenBank. Furthermore, Iraqi sequences were compared to global and vaccine strains of IBDV.

## Methods

### Ethical approval

The study was conducted according to ethical guidelines approved by the committee of ethical approvals of Faculty of Veterinary Medicine, University of Kufa, BEC-20 in Jan_2019.

### Sample collection

A newly died bird from an outbreak of IBDV from a flock in An-Najaf province, was post-mortem examined and the enlarged Bursa of Fabricius was incised and examined.

### RNA extraction

Bursa contents was sampled on (FTA) card (Whatman® FTA® card technology) with four sample areas per card containing cell wall lytic enzymes, protein denaturing agents and inhibit the nucleases effects on nucleic acids ( Ali *et al.*, 2017).

RNA samples on the FTA card were then sent to AniCon® Labor GmbH (Muehlenstraße 13a 49685 Hoeltinghausen, Germany), where the extraction of the IBDV RNA from FTA card was performed using Kylt® RNA/DNA Purification Kit according to the manufacturer’s protocol.

### Sequencing of targeted gene region of *VP1* and *VP2* gene and assigning GenBank accession number

Reverse transcription, PCR amplification and Sanger sequencing of targeted regions of *VP1* gene and hvVP2 were performed in AniCon® Laboratories. The partial *VP1* and *VP2* gene sequences performed in this study were deposited in the GenBank database under accessions MW020533 and MW020534, respectively.

### VP1 and hvVP2 Sequences acquisitioned from GenBank

BLASTn search ( Altschul *et al.*, 1990) was performed against the NCBI database to show the closest matches to the sequenced ones in this study. NCBI BLASTn search results of partial VP1 and partial VP2 sequences and other sequences of vvIBDV from other countries were obtained from GenBank repositories (Extended Data: Table 1) ( Abbas, 2021).

A similar approach was used to get sequences for hvVP2. Sequences of the *VP2* gene of known vaccine strains routinely used in the preparation of vaccines against IBDV worldwide, and available sequences of hvVP2 from Iraq, were also downloaded (Extended Data: Table 1).

### Multiple sequence alignment and sequence manipulation

The IBDV VP1 targeted region obtained in this study was aligned with other similar sequences downloaded from GenBank repositories using MUSCLE ( Edgar and Edgar, 2004), alignments were manually edited, gaps removed and the percentage of pairwise sequence similarity matrices (Extended data: ABIC_andVentriVaccin_IraqV2_DNA_percentSimilrity_matrix.csv) ( Abbas, 2021), were generated using the UGENE pipeline version 35.1 ( Okonechnikov *et al.*, 2012). This was also conducted for the hvVP2 nucleotide sequences.

### Inferring the evolutionary history and time tree

The evolutionary history was obtained by the neighbour-joining (NJ) method ( Saitou and Nei, 1987), with 1000 bootstrap replicates ( Felsenstein, 1985), while the evolutionary pairwise distances (Extended data: IBVD_VP1_NCBI_Hits_OurVP1.txt), ( Abbas, 2021), were calculated by Maximum Composite Likelihood Method (MCL) using Tamura-Nei model ( Tamura and Nei, 1993).

Heuristic search of initial tree was obtained automatically by applying Neighbour-Joining and BioNJ to a matrix of pairwise distances estimated by the MCL method, then the topology was selected with superior log likelihood value.

For hvVP2 sequences of Iraq and vaccine strains, a time-tree to the NJ phylogenetic tree was inferred using the RelTime method ( Tamura *et al.*, 2012; Tamura, Tao and Kumar, 2018). The time-tree was estimated using 31 correction restraints and all ambiguous positions were removed for each sequence to give a final dataset of 309 sites. Molecular phylogenetic analysis was also performed using the maximum-likelihood method based on the Tamura-Nei model ( Tamura and Nei, 1993).

All DNA sequence analyses and evolutionary inferences were performed using MEGAx software version 10.1.8 ( Kumar *et al.*, 2018) and the plotting of datasheets to the phylogenetic tree was achieved by iTOL web application ( Letunic and Bork, 2019) (Extended data: TextFileToShow_GRADIENT_iTol_VP2) ( Abbas, 2021).

## Results

Using molecular methods, we analysed the sequences of the *VP1* gene and hvVP2 sequenced in this study, and other sequences conducted in previous studies in order to assign the IBDV in Iraq to its pathogenic group and predict the best vaccine strain(s) that could be used to evoke the highest possible protection level against IBDV.

### Sequencing and phylogeny of VP1 targeted region

**Figure 1.**
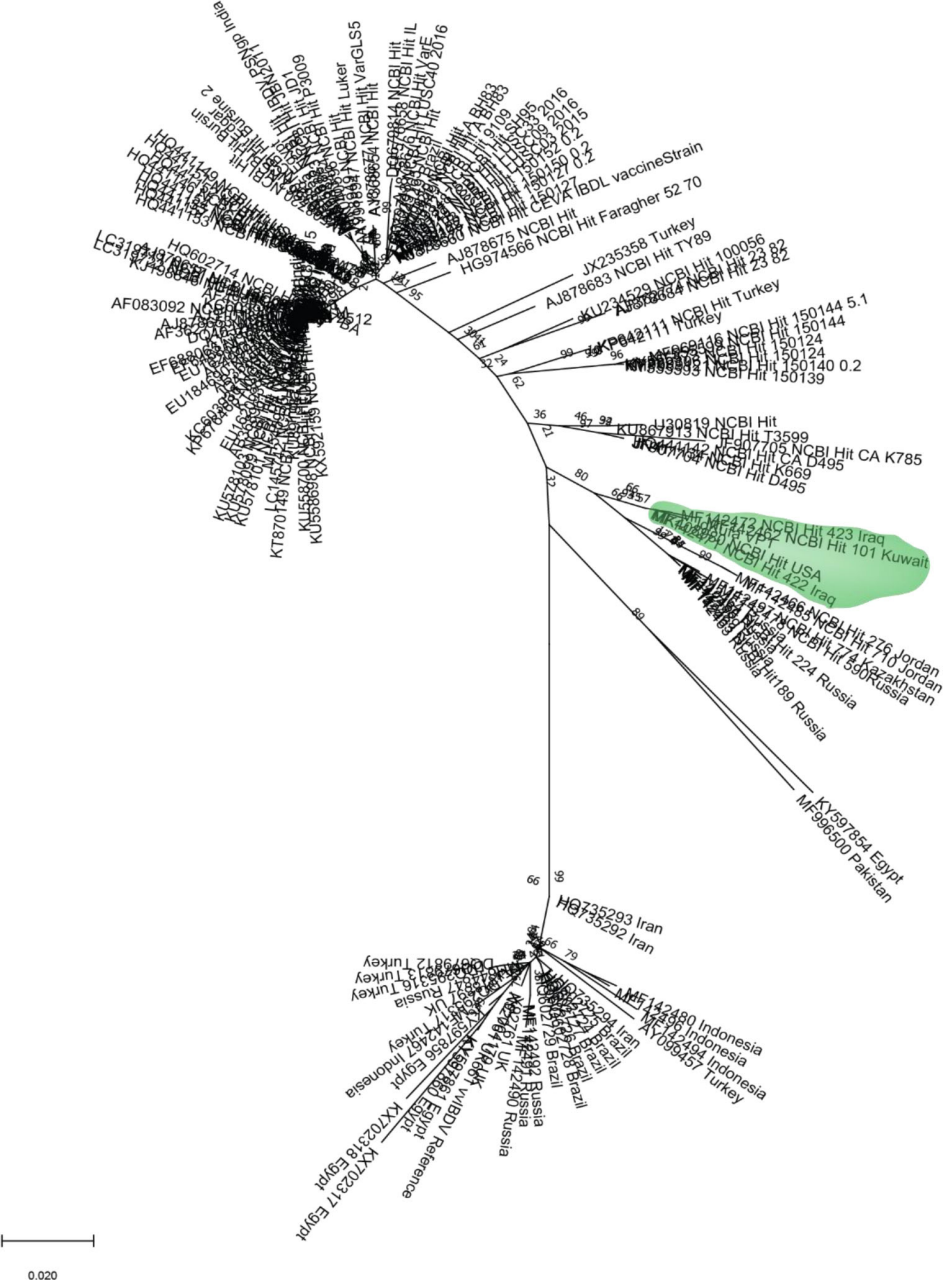
Unrooted phylogenetic tree of VP1 gene. The partial VP1 sequence achieved in this study was clustered in a branch with two sequences from Iraq, one sequence from Kuwait and one from New York, USA. Those sequences were sequenced in previous studies (shaded in green colour). The evolutionary history was inferred by using the Maximum Likelihood method and Tamura-Nei model ( Tamura and Nei, 1993). The bootstrap consensus tree inferred from 1000 replicates ( Felsenstein, 1985) is taken to represent the evolutionary history of the taxa analysed. Branches corresponding to partitions reproduced in less than 50% bootstrap replicates are collapsed. The percentage of replicate trees in which the associated taxa clustered together in the bootstrap test (1000 replicates) are shown next to the branches. Initial tree(s) for the heuristic search were obtained automatically by applying Neighbor-Join and BioNJ algorithms to a matrix of pairwise distances estimated using the Tamura-Nei model ( Tamura and Nei, 1993) and then selecting the topology with superior log likelihood value, the tree was drawn to scale. This analysis involved 145 nucleotide sequences. Codon positions included were 1st+2nd+3rd+Noncoding. There were a total of 528 positions in the final dataset. The phylogenetic data analyses were performed using MEGAx platform ( Kumar *et al.*, 2018) (Extended data: IBVD_VP1_NCBI_Hits_OurVP1.txt) ( Abbas, 2021).

A 544 bp targeted fragment (244-787 bp) of *VP1* gene was sequenced. This sequence, along with other sequences that showed similarity by BLASTn search, were aligned and a phylogenetic tree was constructed using NJ method to reveal the relatedness of the isolated sequence to the existing genogroups/pathogroups of IBDV, regional and worldwide. The VP1 region sequenced in this study clustered in a monophyletic group with strains belonging to vvIBDV genogroup 3: a strain isolated from Kuwait, two strains from Iraq, and one from New York, USA ( Figure 1). The analysis also showed that the most similar sequences from neighbouring countries other than Kuwait were isolated from Jordan.

### Sequence analyses and phylogeny of hvVP2 targeted region

**Figure 2.**
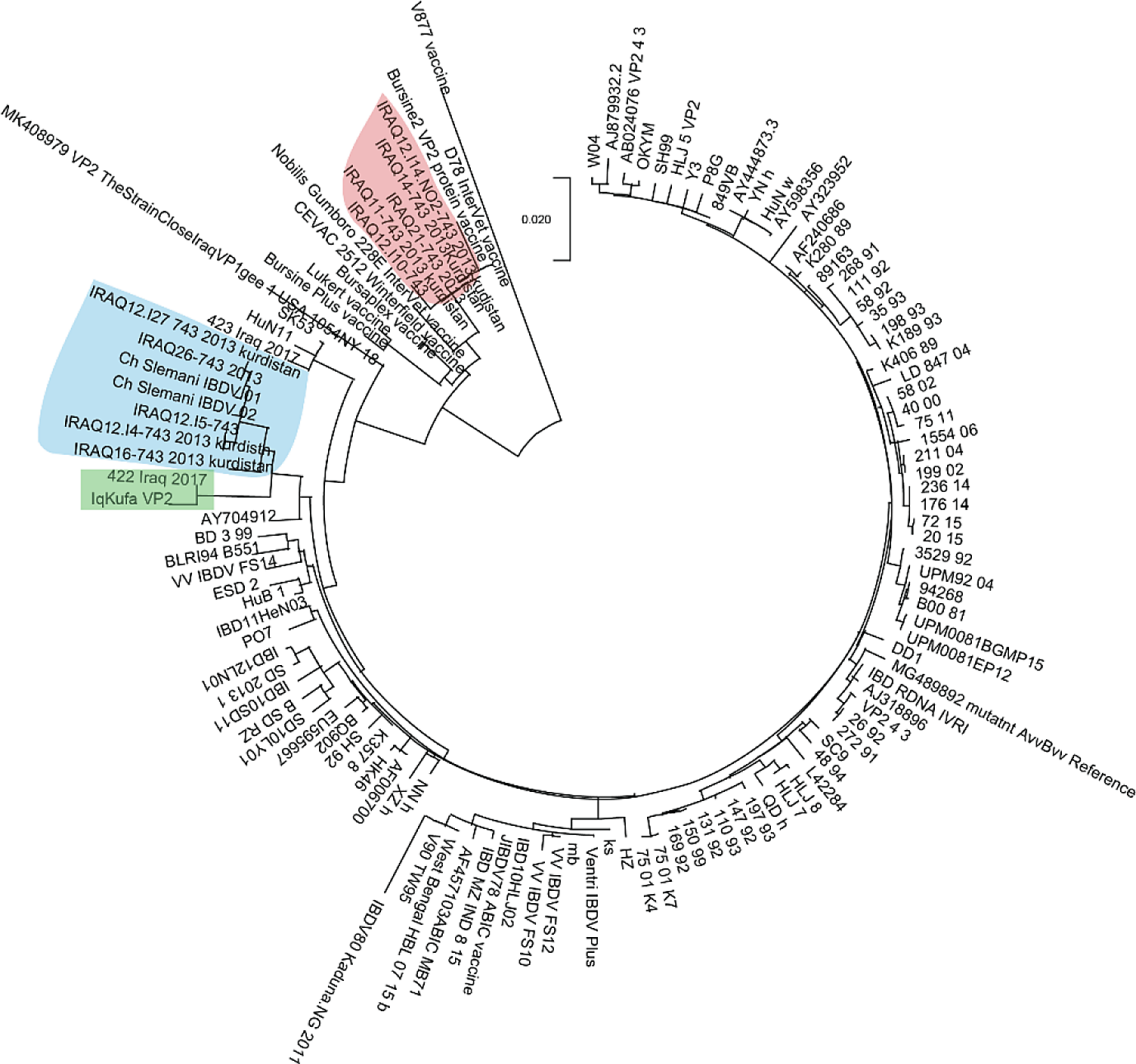
Phylogenetic analyses of hvVP2 region of the sequence conducted in this study with other sequences worldwide. Our sequence was clustered in a single branch with other Iraqi sequences collected in 2017 from previous study (green shaded), while other sequences were clustered in different branches. Remarkably, a group of sequences from the north of Iraq were clustered with vaccine strains (red shaded). The evolutionary history was predicted using the Maximum Likelihood method and Tamura-Nei algorithm ( Tamura and Nei, 1993). The tree with the highest log likelihood (−11351.20) is shown. The tree was drawn to scale, with branch lengths measured in the number of substitutions per site. This analysis involved 122 nucleotide sequences and a total of 3,261 positions were evoked in the final dataset.

A 522 bp segment of hvVP2 region between positions 673-1194 bp of *VP2* was amplified and sequenced. BLASTn search against NCBI repositories revealed high similarity to sequences of the vvIBDV *VP2* gene. Sequence alignment and phylogenetic clustering showed that our sequence paired in a monophyletic group with a sequence isolated from Iraq in 2017 ( Figure 2, green shaded). On the other hand, a cluster of sequences from Kurdistan region in northern Iraq (blue shaded) were branched from our sequence ( Figure 2). These sequences were isolated in previous studies in 2012. However, a sequence isolated from Iraq in 2017 (named 423) was clustered with others from China, and others isolated from Kurdistan ( Figure 2, red shading) were clustered with sequences of vaccine strains ( Figure 2). Such discrepancies shed light on possible genetic reassortments and mutations in the genome of vvIBDV, especially in an important region that acts as an epitope and therefore interacts with antibodies against the virus.

### Sequence divergence and molecular clock of Iraqi VP2

**Figure 3.**
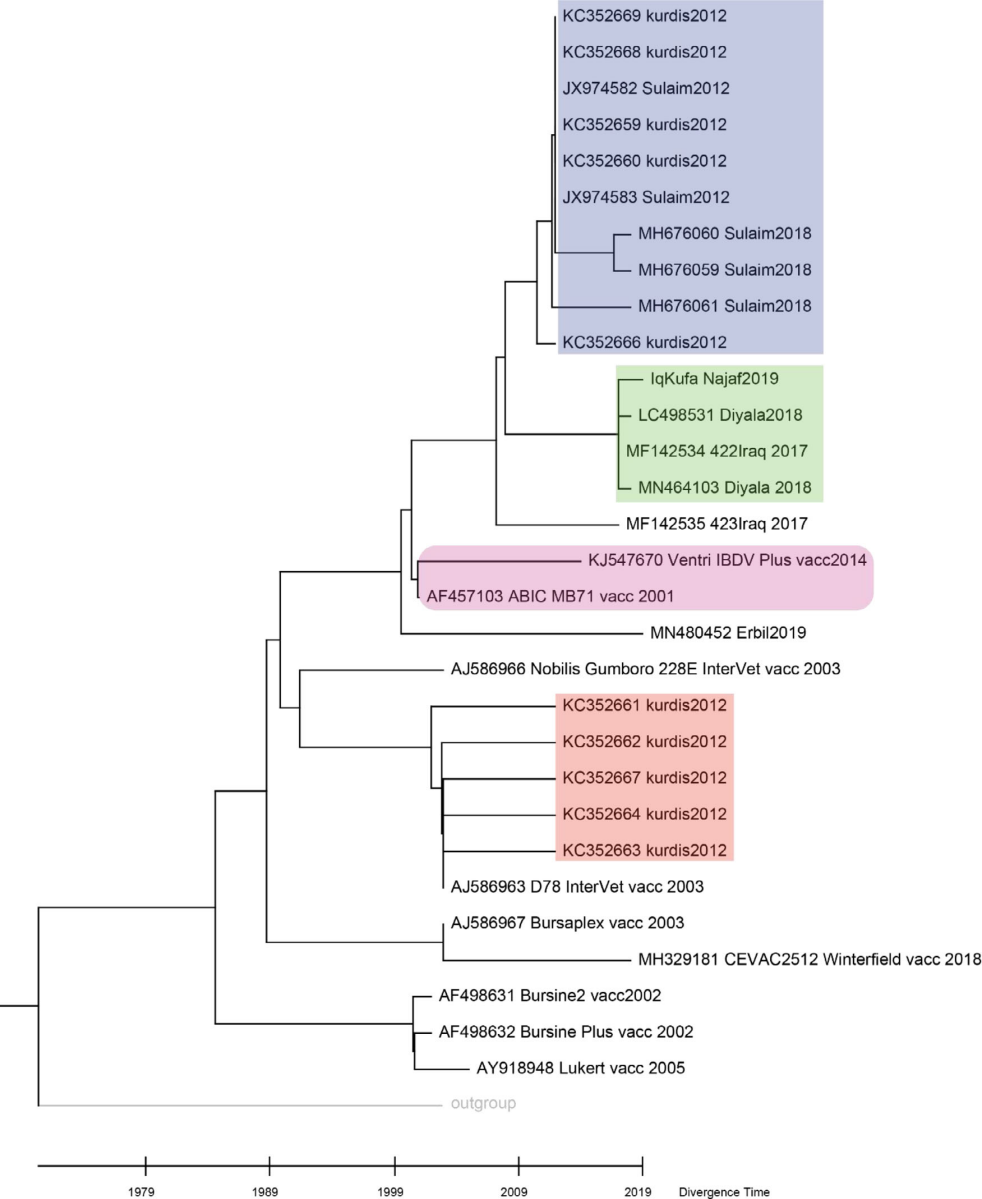
Molecular clock and time tree of sequence divergence of hvVP2 region of Iraqi sequences and vaccine strains. The molecular time tree was computed using 31 calibration constraints on a phylogenetic tree constructed using Neighbour-Joining method. This analysis involved 31 DNA sequences. All ambiguous sites were omitted for each sequence pair (pairwise deletion option). There are a total of 309 locations in the final dataset. Evolutionary analyses were performed using RelTime ( Tamura *et al.*, 2018) in MEGA X ( Kumar *et al.*, 2018). The tree was scaled to the molecular divergence time. In general sequences from central Iraq were clustered in a monophyletic group (shaded in green colour), while those of the Kurdistan region showed different grouping (blue and red colour shading).

The putative sequence divergence proposed by previous analyses motivated us to challenge the possibility of the presence of sequence divergence in the *VP2* gene within strains. NJ phylogenetic tree grouped our sequence with other sequences from Diyala in central Iraq isolated in 2018 and 2017 ( Figure 3, green shaded), while other sequences from Kurdistan, sequenced in 2012 and 2018, were clustered separately ( Figure 3, blue shaded). However, a group of sequences from Kurdistan isolated in 2012 clustered with a vaccine strain D78 Intervet ( Figure 3, red shaded). Such clustering may indicate an origin of infection from a vaccine strain ( Figure 3).

**Figure 4.**
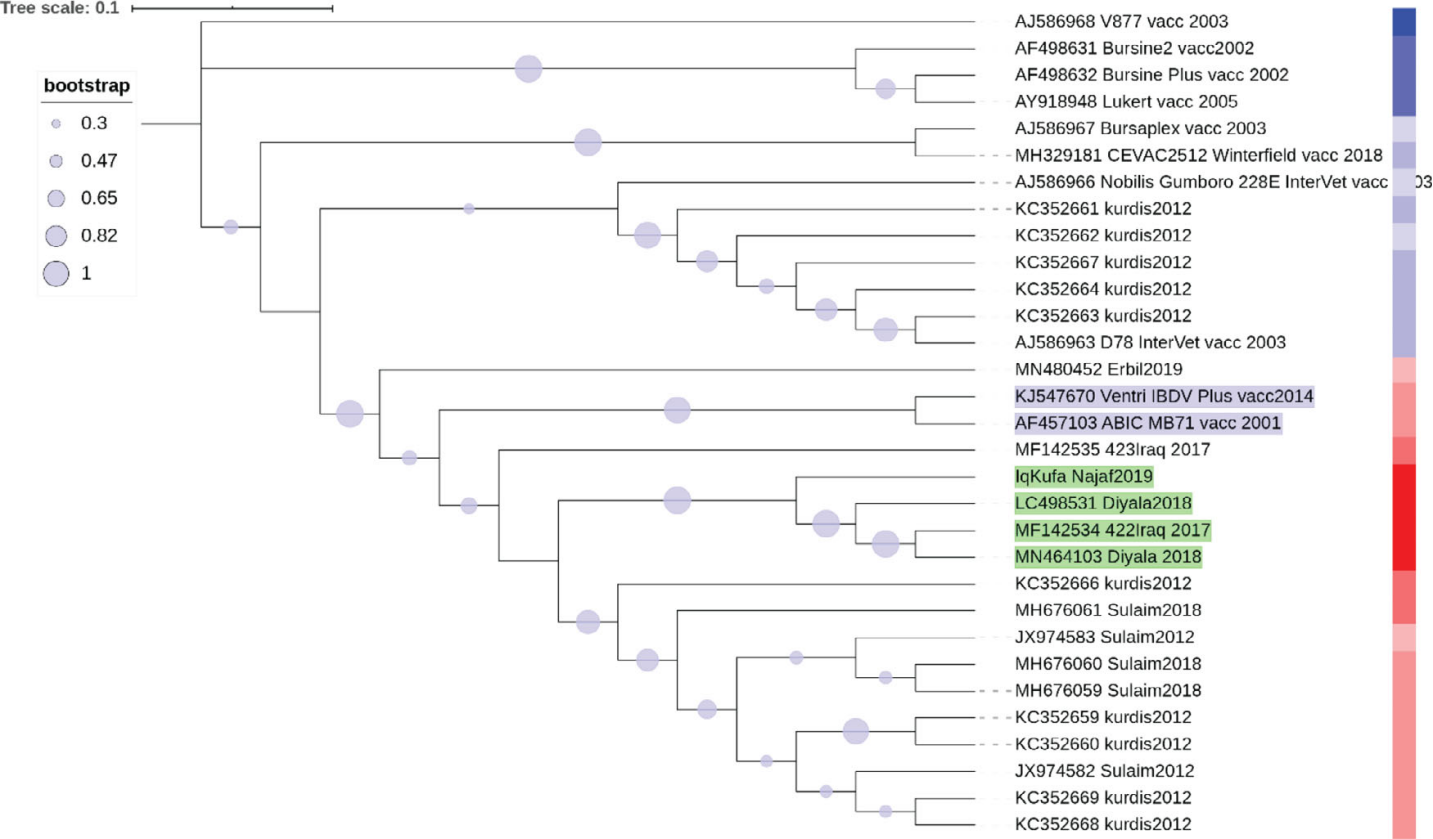
A NJ phylogenetic tree of hvVP2 of Iraqi IBDV strains and sequence similarity heatmap to known vaccine strains. The evolutionary history was inferred by means of the Neighbor-Joining method ( Saitou and Nei, 1987). The optimal tree with the sum of branch length was 0.38665371 was generated. The replicate test of trees in which the associated sequences clustered together in the bootstrap test of 1,000 replicates are shown. Maximum Composite Likelihood method was used to infer the evolutionary distances and are in the units of the number of base substitutions per site. This analysis involved 31 nucleotide sequences. Ambiguous positions were removed for each pair of sequences. The final dataset contains 309 positions. A heatmap of sequence similarity matrices was plotted using web version of iTOL software ( Letunic and Bork, 2019) (Extended data: TextFileToShow_GRADIENT_iTol_VP2), ( Abbas, 2021). The heatmap demonstration of DNA sequence similarity plotted colour coded column next to the sequences names as red (the most similar) to blue colour (the least similar). The closest vaccine strains to Iraqi sequences are shaded in blue.

Hence, it is worthwhile to predict the time these different groups diverged. The molecular clock analyses suggest that the divergence of the sequences from central Iraq and those of Kurdistan region occurred in 2008. Interestingly, sequences from Kurdistan which clustered with vaccine strains showed a divergence time similar to that of a live attenuated vaccine strain D78 Intervet ( Figure 4).

### Prediction of the closest vaccine strain to the Iraqi IBDV local strains

In order to assess which vaccine strain or strains have the most similar hvVP2 sequence to the Iraqi vvIBDV strains, a phylogenetic analysis and DNA sequence similarity analysis approach was conducted. We speculated that such an approach could predict the best vaccine strain that might be applied in control measures against IBDV.

**Figure 5.**
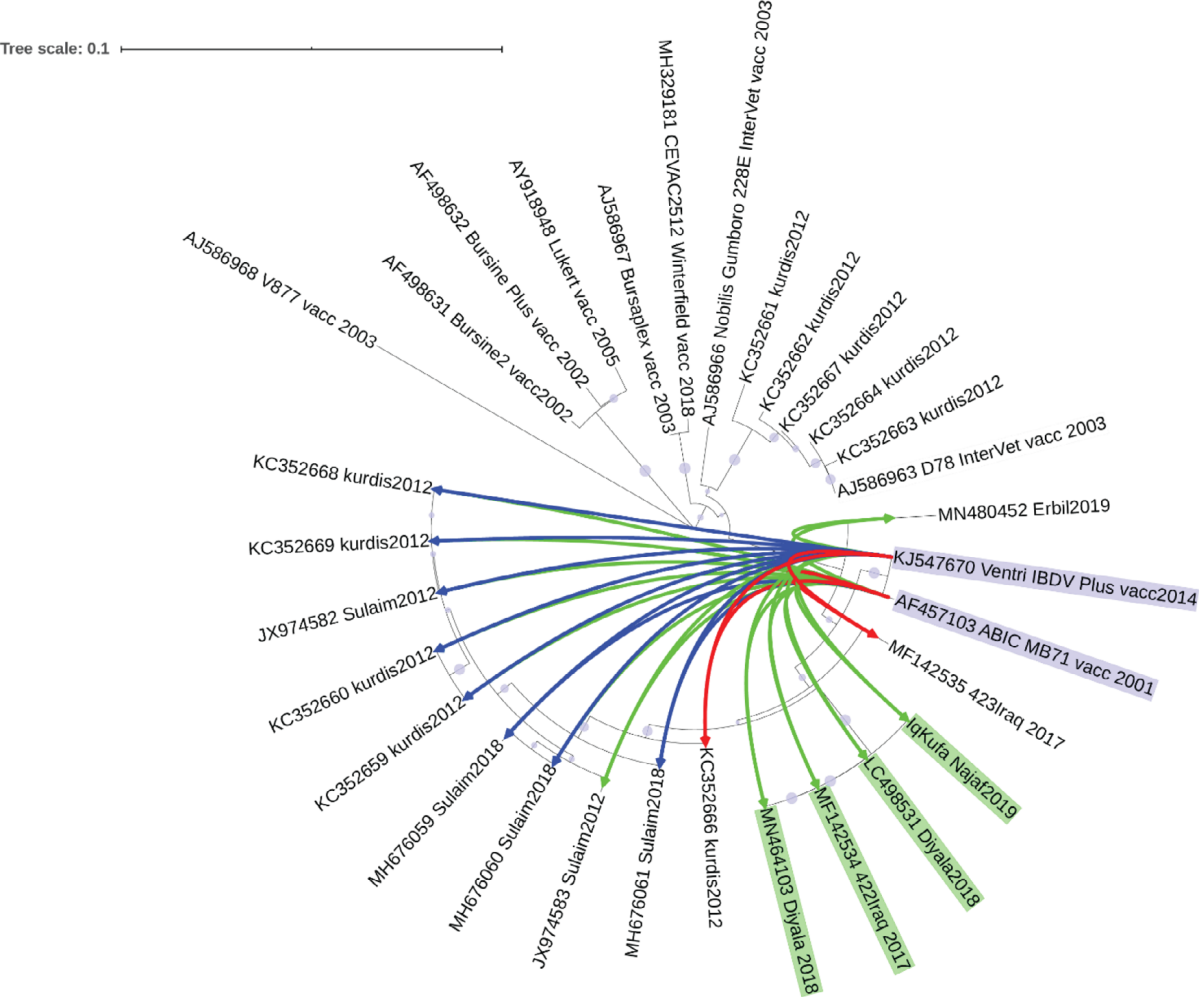
High sequence similarity between hvVP2 sequences from Iraq and two vaccine strains. Sequences of VP2 of vaccine strains Ventri IBDV Plus and ABIC MB71 (blue shaded) showed =>96% sequence similarity (connection lines) to the most sequences from Iraq. Green lines indicating 96% of sequence similarity shown in, blue lines indicating 97% similarity and 98% (red lines) (Extended data: TextFileToShow_connection_iTol_VP2) ( Abbas, 2021). iTOL web application ( Letunic and Bork, 2019) was used to draw sequence similarity data matrix as radial connection lines (values of 96% and above are only shown) to a NJ phylogenetic tree.

This analysis determined that the highest sequence similarity to Iraqi hvVP2 was found in two vaccine strains named Ventry IBDV Plus and ABIC MB71. This showed by both phylogenetic analysis of NJ tree clustering ( Figure 4) and the sequence similarity of more than 96% ( Figure 5).

## Discussion

IBD is a major hurdle in the poultry industry worldwide. Besides its huge direct economic impact, indirect losses are considerable as it reduces productivity and makes entire flocks vulnerable to a number of serious illnesses ( Cosgrove, 1962; Van Den Berg, 2000; Eterradossi and Saif, 2017; Michel and Jackwood, 2017). The most effective way to control this disease is via vaccination. Although vaccination programs are applied, infection with IBDV is still frequent ( Mahgoub, 2012). Iraq is no exception – despite vaccination programs, there continues to be a high infection rate in poultry. However, studies that involve sequencing of targeted regions of IBDV or comparison to the vaccine strains are limited. Therefore, we conducted sequencing of targeted regions of important IBDV genes related to the virulence of the virus ( *VP1* gene), as well as a comprehensive sequence analysis of *hvVP2* region that encodes for the viral epitope that evokes immune response against the virus, using available Iraqi sequences and known vaccine strains.

The phylogenetic analysis of *VP1* gene conducted in this study revealed that our sequence clustered with vvIBDV sequences from previous studies ( Michel and Jackwood, 2017; Michel and Jackwood, 2019). This finding suggests that the IBDV strain sequenced in this study belongs to a vvIBDV based on molecular evidence ( Islam *et al.*, 2012).

In order to identify which genogroup this isolate belongs to, phylogenetic analyses of hvVP2 region were conducted. This analysis showed that our sequence grouped with other vvIBDV sequences belonging to genogroup 3. However, a sequence from Iraq is the only sequence that shared the same monophyletic group with our isolate, while the sequences from Kuwait and New York City seen in VP1 phylogenetic analysis did not show high similarity with our sequence in this genomic region, suggesting mutations are frequent in the hvVP2 region, as seen in previous studies ( Michel, Kimber and Jackwood, 2019).

This result suggests a genetic reassortment. Such genetic modification is not uncommon: previous studies refer to similar genomic rearrangements in IBDV (Ibdv, Jackwood and Sommer-wagner, 2011; Michel and Jackwood, 2019). Other hvVP2 sequences from Iraq, especially those isolated from Kurdistan region (North of Iraq), showed a dispersal aggregation in the phylogenetic tree, suggesting genetic drift and possible mutations affected this protein coding region ( Ali Khan *et al.*, 2019). Despite this sequence variation between the results of the VP1 and hvVP2 phylogenetic analyses, both genic regions were clustered with vvIBDV isolates across the world, suggesting that our isolate is a vvIBDV strain.

Sequences of the hvVP2 region isolated from Iraq have shown a diverse clustering ( Figure 3). While sequences from central Iraq clustered in the same clade, sequences from Kurdistan clustered in different clades indicating possible sequence divergence. Interestingly, a group of sequences from Kurdistan region were clustered with vaccine strains ( Figure 3), especially vaccine strain D78 Intervet, which is used in the preparation of a live attenuated vaccine against Gumboro ( Owoade *et al.*, 2004; Arnold *et al.*, 2012). It has been shown that the live attenuated vaccines may cause pathogenic and clinical manifestations in Bursa of Fabricius ( Müller *et al.*, 2012). This result might suggest either an infection of Bursa of Fabricius by the vaccine strain or it might propose a misdiagnosed infection of the flock, as sometimes live attenuated vaccines lead to clinical signs of infection with Bursa of Fabricius ( Müller *et al.*, 2012; Camilotti *et al.*, 2016). The classification based on sequencing of viral strains is crucial to track the evolution and changes in virulence or antigenicity of such pathogens ( Jackwood *et al.*, 2008; Ibdv, Jackwood and Sommer-wagner, 2011).

Identifying the sequences of field strains and comparing them to the available vaccine strains is an important step in order to employ the most suitable vaccine strain in immunization programs against the circulating local virulent strains of IBDV. The analyses conducted in this study determined that the two vaccine strains (Ventri IBDV Plus and ABIC MB71) have the highest sequence similarity of viral epitope to the local virulent strains ( Figure 4 and Figure 5). However, these vaccine strains have not yet been introduced in vaccination programs in Iraq. Sequence diversity of hvVP2 and lack of evidence of which vaccine strains show similarity to local circulating strains might explain why vaccination programs are failing in many parts of the country.

In this study we showed the diversity of the hyper variable region in the *VP2* gene of vvIBDV and identified the potentially most suitable vaccine strains that could be used in vaccination programs to tackle this major issue in poultry industry in Iraq. However, a wider study involving the collection of many samples from outbreaks of IBDV across different parts of the country is needed in order to map the strains circulating in different regions and track possible sequence changes in the future.

## Conclusion

Although the vaccination program is routinely applied, poultry infection with IBDV is common. In this study we isolated and sequenced a very virulent strain of Gumboro, conducted an unprecedented comprehensive DNA sequence analyses of all available vvIBDV sequences from Iraq, and compared these sequences to vaccine strains.

Our results indicated that sequences of vvIBDV from Iraq belong to genogroup 3 and the antigenic determinant of this virus is prone to genetic mutation, leading to sequence diversification. It is noteworthy that the vaccine strains that revealed the highest sequence similarity to the local virulent strains are not employed in the vaccination programs in Iraq, which might suggest why most vaccinations against IBDV in Iraq are not very effective. Indeed, a wider study involving isolation and sequencing of vvIBDV isolates from different regions across the country is crucial to draw a high-resolution map of the sequence diversity of this virus in Iraq.

## Authors’ contributions

Sequence analyses, interpretation of the results and writing of all versions of the paper were performed by Abbas A.H. Forkan Al and Haider A, conducted the field trips and sample collection. All authors revised the final versions of the manuscript.

## Data availability

### Underlying data

GenBank: Infectious bursal disease virus isolate IqKufa 01 VP2 gene, partial cds, Accession number MW020533.1: https://www.ncbi.nlm.nih.gov/nuccore/MW020533.1.

GenBank: Infectious bursal disease virus isolate IqKufa 01 VP1 gene, partial cds, Accession number MW020534.1: https://www.ncbi.nlm.nih.gov/nuccore/MW020534.1.

Dryad: Supplementary Information for: Sequence diversity and evolution of Infectious Bursal Disease Virus IBDV in Iraq. https://doi.org/10.5061/dryad.s7h44j167 ( Abbas, 2021).

This project contains the following underlying data:

• Table 1_accesson_numbers_of_VP1_and_VP2_sequences_used_in_this_study. (GenBank accessions of VP1 and VP2 sequences used in this study.)
• IBVD_VP1_NCBI_Hits_OurVP1.txt. (DNA sequences of all VP1 sequences used in this study.)
• ABIC_andVentriVaccin_IraqV2_DNA_percentSimilrity_matrix.csv. (hvVP2 sequence similarity percentage of two vaccine strains compared to all Iraqi isolates.)
• all_VP2_DNA_sequencesUsed.txt. (DNA sequences of all VP2 sequences used in this study.)

### Extended data

Dryad: Supplementary Information for: Sequence diversity and evolution of Infectious Bursal Disease Virus IBDV in Iraq. https://doi.org/10.5061/dryad.s7h44j167 ( Abbas, 2021).

This project contains the following extended data:

• TextFileToShow_connection_iTol_VP2. (Connections datasets allow the drawing of straight or curved lines between any two nodes in the tree. Width, colour, and opacity can be set for each line).
• TextFileToShow_GRADIENT_iTol_VP2. (In gradient datasets, each ID is associated to a single numeric value which is converted to a coloured box based on the gradient defined).
• IraqiVP2_pairwiseDistance.csv. (Pairwise distance of VP2 aligned region among strains isolated from Iraq).

Data are available under the terms of the Creative Commons Zero “No rights reserved” data waiver  (CC0 1.0 Public domain dedication).
