# Supplementary material for: Sequence diversity and evolution of infectious bursal disease virus in Iraq
Source: F1000Res. 2021 Sep 2;10:293. Originally published 2021 Apr 16. [Version 2] doi: 10.12688/f1000research.28421.2 (PMC8436185; doi:10.12688/f1000research.28421.2)
Supplement: Peer review history

# Peer review: Reviewer response for version 2

**DOI:** 10.5256/f1000research.76859.r93262

**Published:** 2021-09-10

**Authors:** Daral J. Jackwood

**Affiliations:** 1 Food Animal Health Research Program, Ohio Agricultural Research and Development Center, Wooster, OH, USA; 2 The Ohio State University, Columbus, OH, USA

The author’s revisions are appropriate and improve the paper *, *I am in favor of accepting this revision.

Is the work clearly and accurately presented and does it cite the current literature?

Yes

If applicable, is the statistical analysis and its interpretation appropriate?

Not applicable

Are all the source data underlying the results available to ensure full reproducibility?

Yes

Is the study design appropriate and is the work technically sound?

Yes

Are the conclusions drawn adequately supported by the results?

Partly

Are sufficient details of methods and analysis provided to allow replication by others?

Yes

Reviewer Expertise:

Molecular Virology

I confirm that I have read this submission and believe that I have an appropriate level of expertise to confirm that it is of an acceptable scientific standard.

# Peer review: Reviewer response for version 2

**DOI:** 10.5256/f1000research.76859.r93261

**Published:** 2021-09-10

**Authors:** Fatma Abdallah

**Affiliations:** 1 Department of Virology, Faculty of Veterinary Medicine, Zagazig University, Zagazig, Egypt

I agree to these modifications. I have no objection to indexing the article and good luck to you all.

Is the work clearly and accurately presented and does it cite the current literature?

Yes

If applicable, is the statistical analysis and its interpretation appropriate?

Not applicable

Are all the source data underlying the results available to ensure full reproducibility?

Yes

Is the study design appropriate and is the work technically sound?

Yes

Are the conclusions drawn adequately supported by the results?

Partly

Are sufficient details of methods and analysis provided to allow replication by others?

Yes

Reviewer Expertise:

Molecular Virology

I confirm that I have read this submission and believe that I have an appropriate level of expertise to confirm that it is of an acceptable scientific standard.

# Peer review: Reviewer response for version 1

**DOI:** 10.5256/f1000research.31452.r89761

**Published:** 2021-07-30

**Authors:** Fatma Abdallah

**Affiliations:** 1 Department of Virology, Faculty of Veterinary Medicine, Zagazig University, Zagazig, Egypt

The authors explore the sequences diversity of IBDV in Iraq and compared it with other local and global sequences from GenBank, as well as, compared to available vaccine strains in Iraq. 

 However, I have significant reservations, as outlined below; please address the following comments in your consideration before acceptance:

1. Page 5, the circular unrooted phylogenetic tree of vp1 is not at all clear; please change the phylogenetic tree into another form as traditional to become better and expressive.
2. Please, you can unite the drawing of the all phylogenetic tree in the manuscript as a traditional form to become clearer.
3. In the discussion section, please explain with what motives you have led to suggest that there is a genetic reassortment in IBDV strain in Iraq because the genetic reassortment is not based on genetic drift in the protein coding region.
4.  In the conclusion section, you cannot judge that the available vaccinations against Gumboro disease in Iraq are enough to prevent the current IBDV infection through sequence analysis of all available IBDV sequence strains in Iraq. Indeed, you are needed to isolate circulating IBDV strains from different outbreaks across different regions of your country and then track evolution changes in these strains through studying the pathogenicity and virulence of these strain in vivo; Thus, you can control whether these new isolated IBDV strains are controlled with these available vaccine strains or not. so, I recommend you adjust your conclusion with your results and discussion. 

Is the work clearly and accurately presented and does it cite the current literature?

Yes

If applicable, is the statistical analysis and its interpretation appropriate?

Not applicable

Are all the source data underlying the results available to ensure full reproducibility?

Yes

Is the study design appropriate and is the work technically sound?

Yes

Are the conclusions drawn adequately supported by the results?

Partly

Are sufficient details of methods and analysis provided to allow replication by others?

Yes

Reviewer Expertise:

Molecular Virology

I confirm that I have read this submission and believe that I have an appropriate level of expertise to confirm that it is of an acceptable scientific standard, however I have significant reservations, as outlined above.

# Response

**Published:** 2021-08-21

**Authors:** Ali Abbas

**Affiliations:** University of Kufa, Iraq

Thank you very much for your valuable comments. Please have a look to the following response to your comments:

 For comment one and two:

 We reformatted the phylogenetic trees in figure 1 and 2 to the standard phylogenetic tree to be replacing the radial and circular format, respectively. However, for figure five it is crucial to keep it as is, in order to show the links between different strains.

 3. We'd liked to emphasise that the similarity between strains of IBDV in VP1 gene does not necessarily have sequence similarity in hvVP2 region. Nonetheless, we've omitted the text to remove the confusion.

 4. I've appended a statement, which illuminates the importance of experimental work to be involved in future studies to assess the efficacy of vaccines against local strains.

 However, a cross country molecular study is important to show the diversity of IBDV to reduce the time, cost and labour to achieve the experimental work.

# Peer review: Reviewer response for version 1

**DOI:** 10.5256/f1000research.31452.r83455

**Published:** 2021-05-07

**Authors:** Daral J. Jackwood

**Affiliations:** 1 Food Animal Health Research Program, Ohio Agricultural Research and Development Center, Wooster, OH, USA; 2 The Ohio State University, Columbus, OH, USA

This paper describes the phylogenetic relationship of a vvIBDV strain identified in Iraq with known IBDV strains from the region and around the world. It is also compared to vaccine strains used to protect against IBD. 

 There are three different phylogenetic tree types Traditional, Radiation and Circle used in this paper. It is recommended that only one type of tree be used throughout the manuscript. 

 In the discussion, the authors indicate that the genetic sequence data of the vvIBDV from Iraq suggests a genetic reassortment. It is not clear from the data why this virus is a reassorted strain. Both genome segments aligned with vvIBDV. Reassorted vvIBDV typically have a genome segment B from a non-vvIBDV strain. That does not seem to be the case here. 

 The New York strain of vvIBDV is not from New York City, it is from the state of New York (see the bottom of page 8).

Is the work clearly and accurately presented and does it cite the current literature?

Yes

If applicable, is the statistical analysis and its interpretation appropriate?

Not applicable

Are all the source data underlying the results available to ensure full reproducibility?

Yes

Is the study design appropriate and is the work technically sound?

Yes

Are the conclusions drawn adequately supported by the results?

Partly

Are sufficient details of methods and analysis provided to allow replication by others?

Yes

Reviewer Expertise:

Molecular Virology

I confirm that I have read this submission and believe that I have an appropriate level of expertise to confirm that it is of an acceptable scientific standard, however I have significant reservations, as outlined above.

# Response

**Published:** 2021-08-21

**Authors:** Ali Abbas

**Affiliations:** University of Kufa, Iraq

**Thank you very much for your valuable comments. Please have a look to the following response to your comments:**

 There are three different phylogenetic tree types Traditional, Radiation and Circle used in this paper. It is recommended that only one type of tree be used throughout the manuscript?

**The phylogenetic trees in figure 1 and figure 2 were reformatted to the standard phylogenetic tree to be replacing the radial and circular format, respectively. However, for figure five it is crucial to keep it as is, in order to show the links between taxa.**

 In the discussion, the authors indicate that the genetic sequence data of the vvIBDV from Iraq suggests a genetic reassortment. It is not clear from the data why this virus is a reassorted strain. Both genome segments aligned with vvIBDV. Reassorted vvIBDV typically have a genome segment B from a non-vvIBDV strain. That does not seem to be the case here?

**We'd liked to emphasise that the similarity between strains of IBDV in VP1 gene does not necessarily have sequence similarity in hvVP2 region. Nonetheless, we've omitted the text to remove the confusion.**

 The New York strain of vvIBDV is not from New York City, it is from the state of New York?

**This has been corrected.**
